# Weaving Webs of Care: Older Men’s Roles in End-of-Life Care for their Wives

**DOI:** 10.1007/s10823-026-09564-1

**Published:** 2026-03-06

**Authors:** Seok Joo Youn

**Affiliations:** Department of Anthropology, Washington University in St. Louis, St Louis, Missouri United States

**Keywords:** Spousal caregiver, Older men, Patriarchy, End-of-life care, Webs of care, Ethnography

## Abstract

As South Korea is undergoing rapid demographic shifts and evolving family structures, spousal caregiving has become increasingly vital in older adult care. This growing trend underscores the urgency for research on older men serving as primary caregivers for their terminally ill spouses. Drawing on six months of ethnographic fieldwork with two caregiving cases in a hospice and palliative care (HPC) unit, this study explores how older male caregivers navigate care within the complex interplay of motivations, gender norms, and intergenerational dynamics. The findings reveal that caregiving among older men is deeply influenced by guilt stemming from their roles as primary breadwinners and retrospective acknowledgment of their wives’ sacrifices. Guilt-driven caregiving manifests in the effort to provide care in the form of atonement. Additionally, older men’s caregiving retains patriarchal characteristics, as they assume managerial rather than hands-on roles, delegating tasks to children, in-laws, and HPC staff by establishing “webs of care.” The components of this care network struggle to define good care. A key contribution of this study is its examination of caregiving as a reciprocal process rather than a unidirectional duty. Older husbands act as primary caregivers, but over time they come to require care themselves because of aging-related vulnerabilities. These findings underscore the need for policies that extend caregiving support beyond the family to ensure a more equitable distribution of care responsibilities. By recognizing caregiving as an interdependent and evolving process, this study contributes to rethinking older adult care as a shared responsibility rather than an isolated family burden.

## Introduction

South Korea, one of the fastest aging countries, will become a super-aged society by 2025, with 20% of its population aged over 65 years (Cha et al., 2024). Over the last decade, both domestic and international researchers have paid particular attention to this fast-paced aging trend and its impact on the economy, politics, public health, and social welfare (Choi, 2016; Nakagawa et al., 2021). The demographic shift is related to South Korea’s rapid and unprecedented social and economic development that not only brought about low fertility and population aging but also changes in the typical structure of families from large families to married couple or single-person households (Anbäcken et al., 2021). This change in the family structure continues to influence caring practices (Andruske & O’Connor, 2020; Heo & Kim, 2023).The tradition in which the oldest son and daughter-in-law take care of their parents is disappearing. According to recent South Korean national surveys on informal caregiving, spouse caregivers have the highest prevalence in providing family care; approximately four out of ten older adults receive assistance with functional limitations from their spouses (Choi, 2021). Although South Korean society is aware that older adult care is not the sole responsibility of the family, the family’s role remains important. In other developed countries, 70% of primary caregivers in older adult care are family (Glauber, 2017; Happich et al., 2022), which is higher in South Korea at 80% (Lee & Lee, 2020). This worldwide trend shows that families continue to play an important role in the caregiving of older adults.

Within families, the role of older spouses as primary caregivers may not represent a new phenomenon, but rather a continuation of longstanding marital roles that are marked by heightened responsibility and intensity in later life (Bertogg & Strauss, 2020; Happich et al., 2022). A significant change in caregiving dynamics has been the growing proportion of men providing care to their wives (Fee et al., 2020). This shift results from the rapid increase in the number of older adult households who live far from their children, which makes it challenging for adult children to provide regular care (Jang et al., 2021). Specifically, adult children in their 40 s and 50 s, called the “Sandwich Generation,” often face practical challenges of “double care” for their aging parents because of competing roles and responsibilities as parents of their own children (Jang et al., 2021). Similarly, children in their 60 s and 70 s may struggle to care for their parents because of their own declining health or financial constraints (Salmannezhad et al., 2022).

Following South Korean societal change, research on caring for older adult spouses in the last decade can be classified into two types of approaches: first, determining how spousal care impacts the caregiving spouse’s well-being and the subsequent needs of policy change to alleviate their burden (Ai et al., 2022; Sezgin et al., 2022; Zhao et al., 2023); and second, focusing on the sex-based differences between men and women in caregiving (Bertogg & Strauss, 2020; Fee et al., 2020; Glauber, 2017; Yan et al., 2024).

Because women are perceived as “ideal” caregivers, the experiences of older men providing care have been neglected from research (Zhao et al., 2023). Studies on caregiving by older adult husbands have mostly explored whether husbands undergo changes in their gender role and identity through caregiving, which is considered a “women’s job” (Calasanti & King, 2007). Some studies have focused on the fact that older men perform the “women’s job” in a “masculine way” in spousal caregiving and argue that they provide caregiving while still maintaining or extending their role and identity as men (Choi et al., 2021). Men approach caregiving as they would manage public affairs. In doing so, they maintain or even extend their masculine identities (Lee et al., 2021; Saito, 2023). However, other researchers have viewed husbands’ care as an experience in which gender role and identity is reconstructed. They emphasize that older men use various strategies beyond their conventional role and identity in the process of caring (Choi et al., 2021; Spaeth et al., 2024; Kleinman, 2009).

In addition, qualitative studies on care for older adult spouses have focused on the aging and changing characteristics of their household structure, experiences shared by husbands and wives during their marital lives (Choi, 2021), and gender dynamics of spouses while caring for each other (Cha et al., 2024; Choi et al., 2021). For instance, Cha et al. (2024) stressed that deeper intimacy and emotional attachment between husbands and wives leads to deeper motivation in caring for spouses. Although there has been an increase in academic discussions on spousal care, there is room for further discussion on its importance for older adult spouses. Politically, although policies related to older adult care exist, they have received criticism that they do not capture the changing dynamics of modern families’ structural characteristics.

Although qualitative studies have explored various aspects of caring for older adult spouses in aging societies, they have a few limitations. First, there is a lack of understanding of the relational aspects of the primary caregiver and other members of the family. Second, studies thus far on the gender role and identity of caring for an older adult spouse have focused on the retention and change of gender role and identity in the caring process, and have not deeply examined the specific context in which such roles and identities are dynamically fulfilled. Finally, previous studies have not focused specifically on older adult caregivers, who are also perceived as potential care recipients. Older male caregivers differ from other caregivers, given not just their declining health but also their insufficient ability to conduct domestic chores compared to older adult women. Moreover, most existing studies analyze home-based caregiving, where families shoulder the physical and emotional labor, leaving the dynamics of care in institutional environments largely unexplored. By examining caregiving in a hospital-based HPC unit, this study reveals how older men negotiate authority and responsibility in a distinct context where masculinity shifts depending on its target and the institutional arrangements. Thus, this study seeks to understand the dual existence of male caregivers as caregivers and care receivers from the relational matrix surrounding them.

### This Study

Korean men aged ≥ 65 years have witnessed significant social, cultural, and environmental changes during the latter half of the 20th century. After the division of Korea into two nations in 1948, and South Korea’s efforts to build a modern capitalist state, the country underwent rapid industrialization and urbanization in the 1960s. These changes positioned men as the primary breadwinners, compelling them to devote most of their time and energy to work and socialize with colleagues outside the home. This shift led to the emergence of a “fatherless” complex, where wives and mothers increasingly assumed the role of family heads. Rather than promoting gender equality, this reinforced traditional gender division and hierarchies (Kimmel et al., 2004).

This period overlaps with the presidency of Park Chung-hee (1963–1979). During this era, South Korea implemented four “Five-Year Economic and social Development Plans,” which advanced national industrialization, fostered the growth of large corporations, and facilitated the development of mass education, public transportation, and media, particularly in urban areas (Kwon, 2024). Nuclear families became the norm, with male workers viewed as economic providers and housewives viewed as dependents. Urbanization accelerated as industrialization created jobs in newly established factory cities, drawing a significant labor force from rural areas. This transformation fundamentally reshaped South Korea’s social fabric, economy, and family structure.

South Korea’s hospice and palliative care (HPC) system developed within this broader demographic context. Whereas home hospice is common in many Western countries, HPC in Korea is predominantly hospital-based and integrated into the national health insurance system through a state-led, top-down process (Park et al., 2019). Access is restricted mainly to terminal cancer patients, accounting for over 99% of all HPC users (Kim et al., 2024), and most patients are older adults. Because HPC beds are concentrated in large urban hospitals, families who access these wards tend to be urban residents and capable of coordinating institution-based caregiving. The hospital-based the caregiving cases described in this study illustrate how institutional settings have become central sites for understanding contemporary spousal caregiving.

Specifically, this ethnographic study focuses on two caregiving cases involving older, working-class husbands who spent most of their lives in manual or small-scale labor sectors shaped by Korea’s industrialization. Both men were long-term wage earners with limited economic security in later life and were retired at the time of their wives’ admission to HPC. Their wives had carried the primary burden of childcare and domestic labor for decades.

In this context, this study examines behaviors and emotions through narratives of older adult husbands, who had lived through the turbulent industrial development era, caring for their terminally ill wives receiving HPC. While focusing on the relational aspect of care, this study situates husbands’ narratives in a relational matrix composed of family caregivers and HPC team members. It highlights older male caregivers’ motivation for caregiving, patriarchal characteristics of care, and their dual identities as caregivers and care recipients within the care network. This study expands the scope of discussions on caregiving in relation to gender roles and norms of good end-of-life care in aging societies.

The research questions guiding this study are as follows: How do older men’s life experiences shape their motivation for caregiving? How is the status and authority of an older adult man as the primary caregiver obtained in relationships with his wife and children? What challenges or frustrations do they face due to conflicts of meaning around care?

### Research Methods, Study Site, and Participants

To explore the experiences and behaviors of husbands caring for their wives, this study employs ethnographic methods that provide an in-depth understanding of social actions through participant observations of language, behavior, and beliefs (Hammersley & Atkinson, 2019).

Ethnography is a method of participating in and observing behavioral patterns while living with the participants for a sustained period and attempting to “deeply” understand the internal context and multi-layered meanings of the behaviors through in-depth interviews (Geertz, 2008). As the concept of family care includes complex situations and relationships, ethnography is critical for understanding the detailed nuances of who provides care, how it is provided, and the meaning of care from a particular social significance. The key methodologies of this research (participant observation and in-depth interviews) mainly took place in the consultation room in HPC units. This article draws directly on participant observation conducted during daily rounds, informal conversations in patient rooms, and routine interactions between husbands, their children, and the HPC team. These observations form the core empirical basis of the two cases analyzed here.

The data presented here come from a larger study conducted in an HPC unit of a university-affiliated hospital in Seoul, South Korea over six months (June–December 2024). Following the enactment of the *Act on Hospice and Palliative Care and Decisions on Life-Sustaining Treatment for Patients at the End of Life* in 2016, South Korea expanded its HPC facilities and the scope of diseases covered. Despite growing demand, access to HPC units remains limited by disease type. As of 2024, over 99% of HPC patients in South Korea had cancer and approximately 80% were aged ≥ 60 (Kim et al., 2024).

The principal researcher, a volunteer with three years of experience in the same HPC unit, recruited participants using convenience and purposeful sampling. Referrals from the HPC team members guided the selection process through purposive filtering based on specific criteria. First, families staying in the HPC unit for more than six weeks were considered, as the maximum hospitalization period under the End-of-Life Care Act is two months in South Korea. Extended stay allowed for a deeper exploration of family dynamics and history. Second, participation required the consent of all family members.

This contribution specifically focuses on two in-depth cases of family caregiving in which the primary caregivers were older adult husbands. Primary caregiver was defined as the family member who provided the most prolonged care and assumed overall responsibility for caregiving, including financial support. In HPC units, this person is typically the legal guardian responsible for the patient’s body after the death. Following this definition, the study focused on husbands who identified themselves as the primary caregivers for their terminally ill wives.

Based on these criteria, two family caregiving cases were selected for analysis: the Kim and Lee families. The Kim family consisted of five members: husband Kim (78 years old), his wife Lim (75 years old with terminal-phase breast cancer), two married daughters (the elder is employed, the younger is a housewife), and a son. The Lee family had six members: husband Lee (81 years old), his wife Choi (76 years old with advanced gastric cancer), two sons, and two daughters.

The HPC unit was staffed by two doctors, one physician’s assistant, 12 nurses, one social worker, two coordinators with nursing experience, 27 hospital-employed agency caregivers, two religious staff members, and ten volunteer workers. Except for one male oncologist and two male volunteer workers, all HPC personnel were women, most in their 30 s to 60s. This gendered composition partially shaped the husbands’ interactions with HPC staff not as a matter of individual personality but through culturally patterned expectations regarding gender, authority, and communication in institutional care settings. In the Korean context, these interactions reflected broader sociocultural and communicative norms rather than idiosyncratic personal traits. The agency caregivers often responded first when the husbands and family caregivers of Lim and Choi needed help with their condition. These individuals worked together as part of a multidisciplinary HPC team. The two doctors included an oncologist who conducted the morning rounds and a family medicine doctor who conducted the afternoon rounds. Doctors and physician assistants checked the overall pain symptoms and monitored medication administration. They also checked patients’ urine drainage bags and assessed their health conditions. The 12 nurses were divided into four teams and worked in three rotating shifts, each lasting eight hours per day. The nurses checked vital signs (pulse, blood pressure, respiration, and body temperature) every four hours, and administered pain-reducing drugs and other medication. Social workers and coordinators were present on site from 8:00 am to 5:00 pm. The 27 agency caregivers were divided into three teams of nine members, each working in three rotating shifts. The agency caregivers saw to the patient’s comfort by changing the patient’s position and actively making physical contact. They handled most of the hands-on care, such as the patient’s excrement, changing the diaper, and regularly applying Vaseline to patients’ dry lips and lotion on their face every morning and evening. They sometimes assisted nurses by inserting a nasogastric tube and removing phlegm. Agency caregivers were also the ones who first responded when the husbands and family caregivers of Lim and Choi needed help with their wives’ (or mothers’) condition. Finally, ten volunteers were assigned in pairs, and each team took charge of one day per week on a five-day rotation schedule.

The study is centered on male caregivers’ narratives, focusing on their interactions with children and HPC team members and attitudes toward end-of-life care. The interviews were conducted in Korean and adopted an overt and emic approach, ensuring that the participants were fully informed of the researcher’s role.

### Human Subject Protections

All procedures complied with the relevant laws and institutional guidelines and were approved by the Human Subjects Institutional Review Board of Washington University in St. Louis (Approval Number: 202402111; Approval Date: April 10, 2024). Written and verbal consent was collected from all participants throughout the data collection process, and interviews were recorded with the participants’ consent. All participants were informed verbally and in writing about the study’s purpose, the voluntary nature of participation, and their right to withdraw participation, and they were assured of confidentiality during the analysis and publication process. Pseudonyms have been applied to all participants, and hospital names have been anonymized.

### Data Collection

This study utilized participant observations, in-depth interviews, and field notes to gather data. Primary data collection took place in the HPC units and focused on caregiving husbands’ narratives and their interactions with family caregivers. Most interactions between the husbands and family caregivers were observed next to the patient’s bedside, often near the extra bed provided for caregivers. The researcher visited each patient’s bedside to observe the interactions in detail.

Data were collected from June 2024 to December 2024. Participant observations were conducted for six to eight hours per day to contextualize the daily lives of family members in the HPC units. The observations covered various settings involving family caregivers and focused on their daily routines, actions, and interactions. Special attention was given to husbands’ attitudes and behaviors. The researcher maintained a detailed field journal to capture explicit behaviors as well as subtle unspoken dynamics.

The researcher conducted daily informal interviews with husbands and other family caregivers to gain a comprehensive understanding of the contextual background of the husband-wife relationships and care-related history. These informal conversations allowed the participants to share spontaneous thoughts and reflections in an unstructured manner. The researcher documented informal conversations through detailed field notes immediately afterward.

In-depth interviews were conducted following written and verbal consent obtained in compliance with the institutional review board regulations. Each participant was interviewed for 30 min to 1 h, up to three times per month in a private setting within the HPC unit. The interviews were audio-recorded with the participants’ consent and later transcribed for analysis. The interviews aimed to capture personal narratives, perceptions, and reflections on the caregiving experiences. A professional translator translated the interview transcripts (conducted in Korean) verbatim into English, and the researcher, a native Korean speaker, reviewed the translations to ensure consistency with the original content. Special care was taken to accurately translate the medical terminology, idiomatic expressions, and emotionally nuanced content to preserve the intended meaning in English.

### Data Analysis

This study’s analysis is grounded in Spradley’s technique, which emphasizes that language serves as a fundamental medium for conveying meaning within society (Spradley, 2016). The collected written field notes, informal interviews, and recorded in-depth interviews were initially transcribed and coded in Korean using MaxQDA Pro software, which supports qualitative data analysis using coding techniques. The researcher identified recurring themes, patterns, and narratives from different data sources. The analysis process was iterative, involving multiple rounds of coding and refinement, to ensure that the findings accurately reflected the complexities of the participants’ experiences. The data were coded based on three primary analytical areas: motivation for care, assignment of care, and male caregivers’ dual roles.

The collected data, including recorded interviews, texts, and field notes – all dated and timed – were translated into English, cross-checked by a professional translator, and reviewed by bilingual colleagues to ensure linguistic accuracy and cultural fidelity. The researcher was responsible for all the stages of data collection, coding, and analysis. To enhance rigor and minimize interpretive bias, continuous reflexive journaling, audit trails, and member checking through informal conversations with the participants during fieldwork were performed. Although no external coders were involved, regular consultations with senior qualitative researchers during the study design and analysis stages provided critical feedback on the methodological transparency and interpretive validity, which strengthened the credibility, dependability, and trustworthiness of the findings. The researcher’s language proficiency and cultural awareness of Korea were instrumental in navigating Korean medical institutions.

## Findings

### “She has cancer because of me”: Motivation for Care.

In both families, the husbands expressed the belief that their wife fell ill because they made her suffer. This causal narrative shaped by the husbands became clearer when examined within the context of each couple’s marital life.

#### Contextual Background of the Kim Family

Kim met his wife, Lim, in their hometown of Mokpo, Jeolla-do, and they got married 51 years ago. At that time, the husband ran a dried seafood business in the local market. When their eldest daughter was 11 years old and their youngest son was four, Kim decided to move the entire family to Seoul. Lim had been working as a bank employee in Mokpo, but at her husband’s persuasion, resigned from her job and moved with him.

After relocating to Seoul, Kim attempted several business ventures, all of which failed. It took nearly seven years for him to establish a stable livelihood by opening a store in the Seoul Fish Market. During this period, their eldest daughter entered university, and their second daughter became a high school student. Throughout these years, Kim was unable to contribute to childcare or household responsibilities as he was entirely focused on his business. The full burden of raising the children and managing the household fell on Lim.

Looking back, Kim often reflected on that time, recalling how his wife, who could have continued her stable career as a bank employee in Mokpo, had to endure hardships because of him. He frequently expressed regret and lamented that she suffered because of him.

**Kim**: Just thinking about that time. Sometimes, I just want to forget it. It was so tough. If I struggled, my family struggled too. So, I just worked like crazy. Before I knew it, the kids had all grown up. We never even went on a proper family trip. My wife must have had such a hard time (*taking care of the household*). I think that’s why she ended up getting sick—because she suffered so much emotionally for being with someone like me.

**Researcher**: That must have been really hard for you, too.

**Kim**: It was tough. I don’t even want to think about it. But I wouldn’t have been able to do it without my wife. That’s why I feel so guilty now. [Looking at and talking to wife] I should have treated you well much sooner than this.

Lim was first diagnosed with breast cancer at the age of approximately 60 years. She visited the hospital after a hard lump was discovered in her left breast and was diagnosed with stage two breast cancer. She underwent left mastectomy along with radiation therapy and was declared cancer-free in 2010. However, the cancer recurred in the spring of 2023, and she was diagnosed with stage three breast cancer with axillary lymph node metastasis.

She immediately began chemotherapy, but after six months of treatment, her condition deteriorated because of the grueling regimen, and the cancer continued to spread rather than improve. Her family recalled that she suffered greatly during the six rounds of chemotherapy. Lim eventually discontinued chemotherapy and focused on home-based management through diet and exercise. However, in early 2024, she resumed chemotherapy for four months, only to find that it was ineffective, as the cancer had further metastasized to the cervical lymph nodes and liver. In July 2024, she was transferred to an HPC unit. When Lim was first diagnosed with stage two breast cancer, Kim deliberately acted as if it was not a serious matter. Although he believed that early-stage breast cancer had a high survival rate, he also thought that she had developed cancer because of the hardships she had endured because of him, something he was not ready to acknowledge at the time:

**Kim**: Back then, we caught it (*her cancer*) early. So, I told myself it wasn’t a big deal. Or maybe, I just wanted to believe that. Now that I think about it, she must have felt so alone because of me. I should have been more attentive, more present, but I wasn’t. I think I just didn’t want to face the truth—that I had made her suffer. So, I brushed it off, pretending everything was fine. The kids resented me for it. They thought I didn’t care about (*their*) mother’s condition. But men of my generation—we’re not good at expressing emotions. Even when we don’t feel that way inside, the words come out differently, the actions come out wrong.

**Researcher**: Looking back now, how did you truly feel at that time?

**Kim**: That’s when everything hit me. The thought of losing her, it terrified me. But instead of facing it, I pushed it away. I convinced myself it wouldn’t happen, that she’d always be there.

**Researcher**: How about now?

**Kim**: [Lets out a long, shaky sigh] Now… now we’re just waiting. Waiting for something I don’t know how to stop. And it’s too late—too late to take back all the times I should have held her hand, should have told her I was sorry, should have shown her how grateful I am for her. Now, all I can do is just sit here and watch time run out.

When Lim first fell ill and underwent mastectomy, Kim felt deep regret and guilt. However, he recalled that his inability to express himself properly often hurt his wife. Looking back, he explained that his dismissive attitude at the time was, in fact, an attempt to hide his remorse over the hardships she endured.

Lim raised their three children single-handedly while supporting her husband. After ensuring that all three children had graduated from university, she began working as an insurance planner to contribute financially to the household. When their eldest daughter got married, Lim provided financial support to help secure a newlywed home and contributed to the wedding expenses of their second daughter and youngest son. Kim acknowledged that without his wife’s unwavering dedication to their family, never spending money on herself, and devoting everything to their household, he would not have been able to maintain his current financial stability.

This sentiment was echoed by their children, who cherished their mother deeply and recognized the sacrifices she had made throughout her life:

**Researcher**: What kind of person is your mother?

**Elder Daughter**: My mother worked at a bank for ten years after graduating high school. She didn’t go to college, but she always said she was just as competent—if not more—than the college educated employees. Maybe that’s why she was always so practical and frugal. I don’t think I’ve ever seen her buy new clothes for herself. She always wore my old clothes, even coats that had become too worn out for me to wear. On the other hand, my mother never allowed my father to wear anything worn out.

**Researcher**: Why do you think your father didn’t want to wear worn out clothes?

**Elder Daughter**: She wanted to present her husband well to his customers. She believed that wearing old or shabby clothes would make a bad impression.

Lim is a devoted mother and wife. Through conversations with her eldest daughter, it became possible to indirectly understand the kind of spouse she was to Kim, as well as the context behind his feelings of guilt toward his wife’s frugal lifestyle, which further deepened the guilt that Kim experienced as he cared for her:

**Kim**: Whenever I brought home some money—even though it wasn’t much—my wife never spent a single penny on herself. If she had bought something for herself back then, maybe I wouldn’t feel so guilty now. But she always made sure the kids had what they wanted to eat. She wore the clothes the kids had outgrown. She was such a diligent and frugal person.

#### Contextual Background of the Lee Family

The sense of guilt experienced while caring for a terminally ill spouse was similarly evident in the case of the Lee family. Lee, the husband, and his wife, Choi, got married in Seoul 58 years ago. The couple met while working in a textile factory in the city, and Lee noted that he was immediately drawn to Choi’s meticulous and composed personality. They both described the birth of their first son shortly after their wedding as the happiest moment of their lives. However, amid this happiness, Lee volunteered to serve during the Vietnam War between 1968 and 1973.^1^ He described military life as well suited to his temperament. Given the financial challenges they faced at the time, the higher pay and benefits provided to veterans and their families by the government were significant motivating factors in his decision to enlist. Lee viewed his service to the country as intrinsically linked to his duty to provide for his family.

During his time in the war, Lee sustained an injury to his leg, which led to a permanent limp in his left leg due to inadequate medical care. Additionally, he developed Parkinson’s disease as a result of exposure to Agent Orange, which caused neurological damage and continued to result in tremors in his hands and feet. Below is an in-depth interview with Lee regarding these experiences:

**Lee**: Back then, when the country called, you went. Everyone was struggling to make their living, and the people I worked with at the factory enlisted. I didn’t really want to go (*to Vietnam*), but I did. That’s just how it was at the time.

**Researcher**: Didn’t you find it hard to be away from your wife and children?

**Lee**: I was worried. I had two kids. How hard must it have been for my wife to look after two boys on her own? But back then, the salary wasn’t bad if you voluntarily enlisted in the war. I hoped that the money would help my family’s life (*be*) a little more comfortable.

**Researcher**: What did your wife say about your decision?

**Lee**: We were both young back then. My wife was so strong and determined, so I trusted her. I still remember she cried but she patted my shoulder and told me to come back healthy and not to worry about the kids. I thought everything would be fine as long as I came back in good health, but here I am, with a leg injury. I ended up causing my wife so much hardship.

Lee’s wife, Choi, worked day and night at a factory to raise their two sons while her husband was away at war. She even brought the children to the factory to care for them. The eldest son recalled this experience:

**Eldest Son**: After school, I would go to the factory where Mom worked. When I arrived, my younger brother would be with her, and the youngest was at home with Grandma. When mom finished work around 9 pm, we would all go home together. Now that I have children of my own, I can only imagine how hard it must have been for her. I didn’t realize it back then.

Choi suffered from severe dementia and stayed in a nursing home from 2019 onward. During a health checkup at the hospital in the spring of 2022, she was diagnosed with stage two colon cancer. She received chemotherapy for ten months, but the cancer had spread to her lungs owing to continuous blood-related metastasis, and she was diagnosed with terminal cancer. In September 2024, Choi was transferred to an HPC unit. Owing to her dementia, she did not recognize her children for a long time. Among them, she could still identify and call her youngest daughter, who she saw most frequently. Although saddened by his wife’s memory loss, Lee felt that it may be a blessing in disguise that she no longer remembered the past:

**Lee**: I wish my wife wouldn’t remember the painful past. Sometimes, I think that maybe if she lost those memories, she would be happier, and maybe that’s why God gave her this disease. Now that I think about it, it feels like my wife ended up like this because she married the wrong person. If I hadn’t injured my leg, if I had a proper job, would she be doing better than she is now? I would rather she not remember the past.

As the interviews show, both Kim and Lee felt apologetic toward their wives for voluntarily or involuntarily neglecting childcare and housework for decades because of their work and/or injuries. They also felt regretful that their wives had already begun to suffer from early symptoms of chronic diseases. These emotions seemed to serve as the primary motivations for spousal caregiving. In both families, the husbands developed the narrative that “my wife became ill because of the hardships she endured.” This causal relationship established by the husbands became clearer through an understanding of the context of the couples’ lives.

### The Patriarchal Character of Older Men’s Care and its Webs

After Lim and Choi were admitted to the HPC unit, many people participated in caring for them, including their husbands, children, and hospice team members. They formed “webs of care” for Lim and Choi according to the intent of primary caregivers Kim and Lee as husbands, fathers, and the patients’ legal guardians. The husbands visited the HPC units at the designated times. Both households arranged schedules to ensure proper caregiving for the two patients. Unlike women, who traditionally assume full caregiving responsibilities, older men tend to actively involve their children in caregiving and effectively utilize their support (Cha et al., 2024).

 Tables 1 and 2 illustrate the caregiving schedules of the Kim and Lee households. Husbands Kim and Lee visited the hospital wards from 8 am to 5 pm Monday through Friday when they had no special commitments. The husbands urged the entire family to follow a regular schedule. The husbands came to see the HPC units as places where they could interact and communicate with their family members.

**Table 1:** Timetable of Kim family members’ care schedule

	Monday	Tuesday	Wednesday	Thursday	Friday	Saturday	Sunday
**Morning**	Daughter 2	Daughter-in-law	Daughter 2	Daughter-in-law	Daughter 2	Son 1 & Daughter-in-law	Son 1 & Daughter-in-law
**Afternoon**							
**Evening**	Daughter 1	Son 1	Daughter 1	Son 1	Daughter 1		
**Night**					Daughter 1		

The Kim family’s eldest daughter and son were unable to participate in caregiving during weekday mornings and afternoons owing to their jobs. However, Daughter 2 and the daughter-in-law filled in during these times. Daughter 1 and Son 1 alternated evening visits to care for Lim. On weekends, Son 1 and his wife visited together, taking responsibility for both morning and afternoon caregiving shifts. The proximity of the HPC unit to Son 1’s residence, as compared to the homes of Daughters 1 and 2, was a significant factor in this arrangement. The schedule was created in consultation with Kim. If an unexpected issue arose, leading to a sudden gap in caregiving, Kim was informed first. Because he visited the hospital daily on weekdays, Kim was most familiar with any changes in his wife’s condition.

In the HPC unit, families are expected to provide companionship and assist with basic comfort care such as feeding, light massage, and toileting when possible. These expectations were communicated informally by HPC staff as part of routine care practice, while also reflecting broader, largely unspoken cultural norms in South Korea that position family members as responsible for bedside and comfort care during long-term hospitalization. These tasks often required significant coordination: the Kim children traveled between 30 min and over an hour to the hospital depending on traffic, rearranging work and childcare responsibilities to meet their father’s expectations for consistent presence at their mother’s bedside.

**Table 2:** Timetable of Lee family members’ care schedule

	Monday	Tuesday	Wednesday	Thursday	Friday	Saturday	Sunday
**Morning**	Daughter 1	Daughter 2	Daughter 1	Daughter 2	Daughter 1	Son 1	Son 1 & Daughter 2
**Afternoon**		Daughter-in-law		Daughter-in-law			
**Evening**					Son 1		
**Night**	Son 2	Son 2	Son 2	Son 2	Son 2		Son 2

A similar division of caregiving responsibilities was observed in the Lee household. The two daughters and daughter-in-law, as full-time homemakers, visited the hospital on weekdays. The two sons were unable to participate in caregiving on weekdays because of their work commitments. Instead, Son 2 visited the hospital after 8 pm, stayed overnight, and left for work the following morning, whereas Son 1 concentrated his caregiving efforts on weekends. The Lee children also reorganized their daily routines to meet these caregiving expectations, with some commuting across the city after work or late at night to ensure that someone was always present with their mother.

The doctors conducted rounds twice daily. Kim considered these interactions with doctors to be the most critical part of the day. He used these opportunities to explain his wife’s symptoms and any significant events to the physicians, seek initial assessments of her condition, and determine whether additional medication was necessary. Determined to respond swiftly to any changes in his wife’s condition, he made special requests to agency caregivers stationed in the ward, and offered additional payments as a gesture of gratitude.

A similar pattern was observed in Lee’s case. Volunteers in the HPC unit worked in teams of two. Their tasks included lymphatic massage, bathing, hair washing, and nail care. Lee visited the volunteer office daily to request various services for his wife. His repeated care service requests occasionally placed the volunteers in uncomfortable situations.

### Contestation Over “Good Care”

The husbands Kim and Lee distributed care responsibilities among family members according to set schedules, as shown in Tables 1 and 2, to supplement care provided by HPC members and caregivers. However, not everyone involved in caring for Lim and Choi shared the same understanding as Kim and Lee about what was considered “good care.” During the caregiving process, differences occasionally arose between Kim’s and Lee’s views on good care and those of their children, HPC team members, and other caregivers.

Before his wife became ill, Kim recalled that she enjoyed going to the sauna and frequently requested bathing services from the volunteers. However, medical staff did not recommend frequent bathing because it could cause fatigue and lower body temperature, increasing the risk of infection. Despite this, Kim continued to ask the volunteers to provide bathing services which they, and other family members, felt was not in the patient’s best interest. The following is a conversation between a volunteer and Kim’s eldest son, regarding his mother’s bathing:

**Volunteer**: If your mother catches a cold, it could be really serious. The doctor also says there’s no need for a bath. But your father requested it again yesterday.

**Eldest Son**: I apologize. I’ve already told my father before, but he keeps saying that mother always liked being clean and enjoyed baths, and he thinks she would be happy if he requested the service. I’ll talk to him again.

**Volunteer**: That’s something when your mother was healthy, but now her condition is not good.

**Eldest Son**: Exactly… I don’t understand why my father insists on it. If she catches a cold, it could lead to serious problems.

Despite the medical staff’s objections, Kim’s continued requests for bathing services and attempts to privately use the agency caregiver to address gaps in care were rooted in his understanding of what constituted “good care” for his wife. Similarly, Lee frequently requested massage services from volunteers whenever he noticed slight swelling in his wife’s legs. However, this became a concern for the volunteers as Choi began to experience bone metastasis and the medical staff advised caution when massaging her legs. For volunteers, politely refusing the requests of Kim and Lee was a significant challenge, as shown by the following excerpt from a conversation with a volunteer:

**Volunteer**: To be honest, if the patient is not feeling well, it’s better not to do it (*the massage*). Even just being touched can be bothersome and exhausting for them. And especially for patients like Choi, who have begun experiencing bone metastasis, we have to be extremely cautious. […] It is really difficult (*to refuse*). Especially Kim—he asks every single day. We’ve been volunteering here for over ten years, and from our perspective, his wife is really in poor condition. I think Kim has a certain idea of what constitutes good care and what doesn’t. But from our position, it’s difficult to simply comply with his requests.

Kim expected family members to adhere strictly to the caregiving schedule. He was deeply concerned about any unexpected gaps in care and, if such gaps were unavoidable, he asked the agency caregiver to take care of his wife. However, this request placed excessive burdens on the agency caregiver, as each of them was responsible for three patients and could not provide individualized care for a single patient. The agency caregiver expressed these difficulties to the HPC team and other family caregivers:

**Agency Caregiver**: We are not personal caregivers, so it’s actually difficult to fulfill requests like asking to stay by an individual patient’s side [*as Kim requested*]. I am responsible for three patients at a time. If I start fulfilling individual requests, I will inevitably neglect the other patients, and I would be held accountable for any accidents that occur as a result. There was an incident before where Kim asked so persistently to be next to his wife, but I didn’t notice another patient’s oxygen tube had come loose until later. It almost caused a serious issue. My heart sank when that happened.

Lee’s persistent requests for massages for his wife, who was undergoing bone metastasis, stemmed from his desire to do something for her, reflecting his belief that such actions were part of providing good care.

**Lee**: Maybe today could be the last day with her. It could be the last day I’m able to do something for my wife. I know I made her suffer when we were younger, but now I just want to do my best for her as she’s nearing the end. So, I’m asking the volunteers to provide massage services now, so she can be relaxed and comfortable.

Both husbands, Kim and Lee, driven by guilt over having caused their wives suffering when they were healthy as well as a sense of responsibility that their wives’ terminal cancer was ultimately a result of their own actions, attempted to alleviate these feelings by outsourcing caregiving responsibilities. This is another example of how caregiving by older adult men can manifest in a patriarchal manner (Mott et al., 2019).

In the HPC unit, the concept of “good care,” as understood by Kim and Lee, sometimes clashed with the views of the medical staff, volunteers, caregivers, and children. Often, the “good care” that the husbands envisioned for their wives was individual and specific, derived from the unique context and experiences of their married lives. In the HPC unit, multiple interpretations of what constitutes good care for the wife, mother, or patient were in competition. Specifically, among the HPC team members, the medical staff defined good care as preventing the patient’s condition from deteriorating, whereas the agency caregivers, who were responsible for looking after multiple patients at once, interpreted good care as providing equitable care for each patient. Thus, the webs of care in the HPC unit were not only a “web of social relationships” but also a “web of significance” (Geertz, 1973), that is, a dense network of meanings through which individuals interpreted and enacted care. Each caregiver—whether husband, healthcare providers, or children—brought their own cultural, emotional, and moral understandings to what constituted good care, creating overlapping, and at times conflicting, frameworks of meaning within the shared space of the HPC unit.

### Caregivers as Care Recipients

Within the webs of care, there was a contest over who could be considered the “care recipient.” Husbands Kim and Lee were both legal guardians of their wives Lim and Choi, bearing financial responsibility for hospital expenses and devoting most of their time and attention to their wives’ care. Both husbands had no doubt that they were the closest individuals to their wives and the “primary caregivers.” Although both husbands viewed themselves as the core “agents of care” for their wives, their children did not perceive them as partners in caregiving but rather as another “care recipient.”

Initially, Kim was perceived as a care recipient by his children. As Lim began her battle with cancer and could no longer manage household responsibilities, the children became increasingly concerned about their father’s meals, second only to their concern for their mother’s health. Because Lim had always been the one to prepare meals, Kim, who lacked basic cooking skills, started to rely on ready-made food bought at convenience stores. Upon discovering this, the children worried about the potential deterioration of their father’s health. As they visited the HPC unit to care for their mother, the children frequently checked on their father’s well-being and ensured that he had proper meals. On weekends, the daughters alternately visited their father’s home and assisted with cleaning and other household chores.

The recognition of Kim as a person in need of care was also shared by the other caregivers. When Kim attempted to reposition his wife, the agency caregivers intervened. Because there was a risk of Lim falling from the bed, the caregivers believed that Kim, owing to his age and physical limitations, would find it difficult to bear his wife’s weight. Similarly, when Lim used a wheelchair to move, the caregivers prevented Kim from pushing it as they thought it would be physically strenuous for him as an older adult. The following is a conversation between the researcher and the agency caregiver:

**Researcher**: Kim wanted to push his wife’s wheelchair himself. Could you explain why you intervened?

**Agency Caregiver**: The wheelchair is quite heavy. because the patient lacks strength in her body, her weight alone is already considerable, and adding the weight of the wheelchair makes it extremely heavy. Also, considering that Kim is an older adult, there’s a risk that he might injure himself or cause an accident while trying to push it. It is better for us to handle it.

A similar pattern was observed in the Lee family. Lee, who had hypertension, diabetes, and Parkinson’s disease, visited the hospital regularly. Additionally, after an incident five years before, when Lee had collapsed at home due to a stroke and was rushed to the emergency room, the family viewed him not only as the primary caregiver but also as a potential patient in need of care.

**Elder Son**: I understand that my father wants to do something for my mother. However, sometimes, it actually makes things worse. Just the other day, he tried to empty mother’s urinal himself and ended up spilling it in the bathroom. It caused trouble for the nurses, agency caregivers, and other patient’s families … He needs to realize that he is also a patient. Just because his physical condition is better than my mother’s doesn’t mean that we can say he is healthy. We are worried that if his condition worsens too, it will only make things more difficult for all of us.

Lee was aware of his family’s concerns, yet persisted in his desire to provide care for his wife.

**Husband Lee**: I’m not lying in a hospital bed like my wife, waiting for death like she is. Both my son and family keep telling me not to do anything. It’s frustrating. I know they’re saying this because they care about me, but it feels like they’re treating me like a patient.

Lee’s statement illustrates the conundrums of being both a caregiver and a recipient within the web of care. The family caregivers often regarded Lee’s efforts to do something for his wife as a source of concern. This issue was particularly pronounced in cases where the caregiver had chronic symptoms. Lee found it challenging to provide practical care for Choi because of Parkinson’s disease, which caused tremors and mobility difficulties. For example, because Choi used a catheter for urination, the urine collection bag was emptied regularly. Furthermore, as she had difficulty maintaining her posture, Lee had to insert a straw into a cup of water and bring it to her mouth whenever she was thirsty, and regularly lift her body to change its position. However, because of Parkinson’s disease, Lee was unable to perform these simple caregiving tasks. This explains why, within the care network, Lee was recognized as someone in need of care rather than as the primary caregiver. This shows that the web of care serves not only terminally ill wives but also older adult husbands. Ironically, continually being within the hospice setting as a caregiver exposed Lee to receive forms of care that would otherwise be absent in his domestic life.

Despite recognizing Lee as someone in need of care, his children did not interfere with his routine hospital visits. They believed that maintaining a regular routine to keep up with the schedule for caring for his wife was beneficial. They also thought that it was advantageous for them to frequently meet and talk with their father at the hospital as it allowed them to check up on him.

**Elder Daughter**: Wouldn’t it be better for him (*father*) to come here instead of staying home alone all day? Here, he can see mom, talk to us, and it’s more beneficial. When he’s here, we can also observe his condition during lunch, see how he’s doing, and understand what he’s thinking these days. By coming back and forth, he also gets to walk around and stay active, which I believe is better for his health. My siblings also think it’s better than just staying at home.

Social activities and participation play a crucial role in enhancing quality of life, restoring self-esteem, and maintaining mental health in older adults (Choi et al., 2021; Hupkens et al., 2018). In particular, studies have emphasized that interpersonal intimacy and human relationships are the most important means of finding meaning in life in old age. The acts of caring for a wife, forming a web of care, and engaging with various people were ways in which husbands Kim and Lee participated in social activities. Their children also recognized this as an essential element for the quality of life and health of their fathers. However, in the HPC units, the webs of care that the husbands had proactively woven paradoxically functioned as a means to care for themselves. This ultimately demonstrated the conflict over the meaning of “who is the recipient of care.” This conflict suggests that caregiving is not a simple, one-way relationship but rather a reciprocal process, and its interpretation can vary depending on social and emotional factors.

## Discussion

This study underscores the intricate motivations and structural dynamics of spousal caregiving in older men in HPC settings. The findings reveal that caregiving by older adult men is not merely an act of duty but an emotionally complex and socially constructed process shaped by personal histories, intergenerational obligations, and institutional care frameworks. The key themes emerging from this study (guilt as a driving force, persistence of patriarchal caregiving structures, contested notions of “good care,” and the dual role of caregivers as care recipients) provide critical insights into the nuanced realities of spousal care in later life. Both cases involve wives in terminal stages of cancer receiving HPC, situating the husbands’ caregiving within an imminent end-of-life context.

By closely examining the dynamics among the caregivers forming webs of care, this study shows that older men’s spousal caregiving is based on their patriarchal character and is simultaneously biographical, hierarchical, and contradictory. These insights contribute to a deeper understanding of the emotional, social, and structural factors shaping the experiences of older male caregivers who, despite aging, continue to navigate traditional gender roles and familial responsibilities.

A central finding of this study is the profound role of guilt in shaping the caregiving behaviors of the men. Kim and Lee perceived their wives’ illnesses as a consequence of the hardships they endured throughout their marriage. This guilt, rooted in their historical roles as primary breadwinners during South Korea’s industrialization, led them to retrospectively acknowledge their wives’ sacrifices (such as giving up careers, raising children alone, and managing household affairs), fueling their commitment to provide care in their wives’ final stages (Mohamed Hussin & Mohd Sabri, 2023). These findings align with research suggesting that men often view caregiving as an atonement rather than as a familial duty (Ofuoma & Ncama, 2024). Unlike women, who traditionally perceive caregiving as an expected responsibility, the male caregivers in this study engaged in care as a means of reconciling past emotional and relational deficits (Huertas-Domingo et al., 2023). This guilt-driven approach often created tension with HPC staff and family members.

It also illustrated how patriarchal norms shape caregiving among older adult men. Despite their involvement, the husbands in this study structured a web of care involving children, in-laws, and HPC staff, rather than assuming full caregiving responsibilities. Unlike women, who typically engage in hands-on caregiving, older adult men acted as managers, overseeing schedules, making decisions, and delegating tasks, aligning with previous research on male caregivers’ administrative approaches to caregiving (Cha et al., 2024). This managerial role reflects traditional masculine identities, in which men maintain authority even in contexts demanding emotional labor and physical care (Choi et al., 2021; Hupkens et al., 2018). At times, the husbands’ managerial authority was not constructed in isolation but continually reinforced, and at times subtly challenged, by their children. However, fathers’ authority was also constrained when children questioned their physical capability or intervened to prevent risky caregiving actions, revealing quiet forms of resistance that expose the limits of paternal authority in later life.

Another key theme is the contested nature of good care in the HPC units. Kim and Lee viewed specific caregiving actions, such as frequent bathing or massage, as essential to their wives’ well-being, whereas the HPC team members often opposed these requests because of medical concerns. This divergence in perspectives underscores the subjective nature of good care, which is shaped by personal histories, cultural expectations, and institutional protocols. For the husbands, good care was informed by their intimate knowledge of their wives’ past preferences and their own desire to provide tangible expressions of love and remorse. However, from a medical standpoint, good care was defined in terms of minimizing physical distress and preventing complications. This tension underscores a broader challenge in end-of-life care, where medical expertise and familial emotional needs often collide and require careful negotiation among stakeholders. Drawing on insights from medical anthropology, such tensions can be understood as clashes between biomedical rationalities and the symbolic and emotional world through which families interpret illness and dying (Good, 1994; Kleinman, 1980). In the focal cases, husbands’ notions of good care emerged less from clinical logic than from culturally rooted obligations and relational meaning-making.

Finally, this study highlights the paradoxical position of older male caregivers as providers and recipients of care within the web of care. Although both Kim and Lee identified themselves as primary caregivers, their children and HPC members increasingly regarded them as individuals in need of care because of their aging-related vulnerabilities. This duality was particularly evident in Lee’s case, where his chronic illnesses limited his ability to provide physical care, leading family members and medical staff to redefine his role within the caregiving web. These findings support the view that caregiving in old age is inherently reciprocal rather than unidirectional. Older caregivers frequently transition from care providers to support recipients; however, they may resist this shift because of personal and cultural expectations surrounding masculinity and autonomy. This finding underscores the fact that the web of care served not only the terminally ill wives but also the older husbands who originally established the web.

Beyond these cases, the findings also speak to broader debates on gender justice and the future of caregiving masculinities in South Korea and other aging societies. Although the older men in this study largely enacted traditional managerial forms of masculinity, their presence in end-of-life care highlights the societal need for more equitable and relational models of male caregiving. Current discussions in Critical Studies on Men and Masculinities suggest that men’s caregiving roles can evolve toward practices grounded in emotional engagement, reciprocity, and vulnerability (Elliott, 2016; Roberts & Prattes, 2024). In the context of rapid population aging and an escalating social care crisis, transforming masculine norms around care is becoming central to creating just and sustainable caregiving arrangements.

The results of this study have academic and policy implications for caring for terminally ill spouses in an aging society. First, it is necessary to consider older adult care within the framework of webs of care. If the patient is relatively healthy, it is enough to provide physical and psychological care by living with the patient or visiting them frequently; however, if the patient is on the verge of death, it is necessary to provide various complex caring behaviors, such as covering expenses for treatment and care, performing the role of legal guardian, making important medical decisions and judgments, and providing actual physical care. To handle these duties, it is necessary to form webs of care that include a fair distribution of roles among caregivers and to create a culture of care.

Second, as caregiving responsibilities become increasingly multigenerational, policies should consider the intersectional dynamics of family caregiving. Specifically, support systems should address the dual caregiving burden faced by adult children who are simultaneously responsible for caring for both older parents and their own children and provide resources to alleviate these pressures.

Finally, it is necessary to establish webs of care beyond families. As shown in this case study, families in Korea have traditionally taken women’s or children’s sacrifices for granted. Hierarchy and discrimination within the family force women and children to yield based on empathy and respect. The case in this study is significant in that it showed the need to consider older adult care in terms of forming webs of care, while also highlighting the limitations of these webs. As all humans depend on the care provided by others at certain points in their lifetime, caregivers and recipients cannot be clearly distinguished. In a population where the composition and family structure are changing, older adults can no longer expect to receive care from their children and must expect to be taken care of by someone other than their family. Thus, equal and reciprocal webs of care must be built within society without relying solely on family members.

### Strengths and Limitations

This study’s primary strength lies in using ethnography to collect information based on the authentic experiences and holistic views of husbands and family caregivers. This approach facilitated a deep and nuanced understanding of the husbands’ motivations, psychology, and attitudes toward end-of-life care. Another key strength is that the institutional setting provides analytic insight into how older men negotiate caregiving within structured medical routines and interactions, which are not accessible in home-based research. Conversely, conducting research in a single hospital-based HPC unit also constitutes a limitation by restricting generalizability. Future research could expand on this work by exploring spousal caregiving experiences across diverse cultural settings, examining non-family-based caregiving models, and investigating the long-term impact of caregiving on men’s identities and well-being.

The study’s other main limitation is that the analysis was conducted primarily by a single researcher. Strategies such as peer debriefing with qualitative research experts, participant feedback on the findings, and regular reflexive discussions were employed to enhance credibility and transparency. Although these measures helped to reduce individual bias, collaborative data analysis with another researcher throughout the process could have further enriched the interpretation and strengthened the findings.

## Conclusion

This study highlighted the complex and deeply emotional nature of older adult spousal caregiving, revealing how guilt, patriarchal caregiving structures, and shifting familial roles shaped the experiences of older male caregivers. Rather than simply fulfilling their duties, these men navigated caregiving through the lens of past sacrifices and relational atonement. Their managerial approach to care, rooted in traditional masculinity, often led them to delegate tasks while maintaining authority, and reinforcing broader gendered caregiving patterns. Simultaneously, their struggle to define good care within HPC units underscored the tension between personal, cultural, and medical caregiving perspectives. These findings reaffirm the reciprocal nature of older adult care, in which aging husbands, initially positioned as caregivers, are also care recipients within the web of care they construct.

Considering South Korea’s rapidly aging population and evolving family structure, this study underscores the urgent need for policies that extend caregiving support beyond family units. The increasing prevalence of spousal caregiving calls for a systemic approach that distributes caregiving responsibilities equitably. Moreover, as caregiving becomes more multigenerational, policies must address the dual caregiving burden placed on adult children and ensure that they receive adequate resources to balance their responsibilities across generations. Ultimately, building sustainable and reciprocal webs of care, both within families and through broader societal support, is essential for addressing the complexities of older adult care in an aging society. Future research could further examine how the imminence of death shapes men’s motivations, emotional labor, and gendered identities in caregiving, offering deeper insight into how traditional masculinities are sustained or unsettled at the end of life.

## Data Availability

The data of this study are available from the author upon reasonable request. Owing to the sensitive nature of the interviews and fieldnotes, and to protect participants’ privacy, access is restricted and will be granted only for legitimate academic or ethical inquiries.

## Footnotes

1 South Korea participated in the Vietnam War from 1964 to 1973, sending over 300,000 troops in support of the United States and South Vietnam. It was the largest foreign contingent after the U.S., with forces primarily engaged in combat, pacification, and reconstruction efforts. In return, South Korea received substantial U.S. military and economic aid, which contributed to its rapid industrialization.
